# Comparative Analysis of the Mitochondrial Genomes of Chloropidae and Their Implications for the Phylogeny of the Family

**DOI:** 10.3390/ijms25052920

**Published:** 2024-03-02

**Authors:** Jiuzhou Liu, Jiajia Chen, Xiaodong Cai, Ding Yang, Xuankun Li, Xiaoyan Liu

**Affiliations:** 1 Hubei Insect Resources Utilization and Sustainable Pest Management Key Laboratory, College of Plant Science and Technology, Huazhong Agricultural University, Wuhan 430070, China; liujz96@163.com (J.L.); jjc184989@163.com (J.C.); 2 College of Plant Protection, China Agricultural University, Beijing 100193, China; caixiaodong1031@163.com (X.C.); dyangcau@126.com (D.Y.)

**Keywords:** Chloropidae, mitochondrial genome, phylogeny, Bayesian analysis, maximum likelihood

## Abstract

Chloropidae, commonly known as grass flies, represent the most taxonomically diverse family of Diptera Carnoidea, comprising over 3000 described species worldwide. Previous phylogenetic studies of this family have predominantly relied on morphological characters, with mitochondrial genomes being reported in a few species. This study presents 11 newly sequenced mitochondrial genomes (10 Chloropidae and 1 Milichiidae) and provides the first comprehensive comparative analysis of mitochondrial genomes for Chloropidae. Apart from 37 standard genes and the control region, three conserved intergenic sequences across Diptera Cyclorrhapha were identified in all available chloropid mitochondrial genomes. Evolutionary rates within Chloropidae exhibit significant variation across subfamilies, with Chloropinae displaying higher rates than the other three subfamilies. Phylogenetic relationships based on mitochondrial genomes were inferred using maximum likelihood and Bayesian methods. The monophyly of Chloropidae and all four subfamilies is consistently strongly supported, while subfamily relationships within Chloropidae remain poorly resolved, possibly due to rapid evolution.

## 1. Introduction

Chloropidae, commonly known as grass flies, is the most taxonomically diverse family of Carnoidea. It currently comprises 203 described genera and over 3000 known extant species worldwide [1,2]. Their bodies are usually mostly black or primarily yellow with black to brown stripes, and they have diverse body shapes, from short and broad to greatly elongated, with their total lengths ranging from 1.0 to 9.5 mm in size [3,4]. The origins of this family coincide with the schizophoran radiation during the early Tertiary [5], and the reported fossils have provided a glimpse into their diversity in the Eocene and Oligocene [6].

Some chloropids are of agricultural or medical importance, including crop pests of grasses and cereals (Poaceae) (e.g., *Chlorops* spp., *Dicraeus pennisetivora*, *Oscinella frit*, *Meromyza* spp., etc.), potential biocontrol agents (e.g., *Thaumatomyia* spp.) [4], and vectors of the pathogens of conjunctivitis and Brazilian purpuric fever (e.g., *Hippelates* spp.) [7,8]. Some species are known pollinators of *Genoplesium* (Orchidaceae) [9], *Aristolochia* (Aristolochiaceae) [10], *Phoradendron* (Santalaceae) [11], and *Ceropegia* (Apocynaceae) [12]. Many chloropid species are saprophagous, meaning that they live in decaying plant tissues or animal carcasses [13], and they play an important role in the decomposition of vegetable matter [8].

The monophyly of Chloropidae is widely accepted [2,4,14,15], as it is strongly supported by several synapomorphic characters: reduced chaetotaxy, developed ocellar triangle, vertical propleural carina, the absence of basal medial crossvein (bm-m) and anterior cubital cell (cell *cua*), and the presence of flexure in the fourth medial vein (M_4_) [14,15]. The Milichiidae is recovered as the sister group of Chloropidae based on molecular and morphological data [5,14,16].

**Figure 1:**
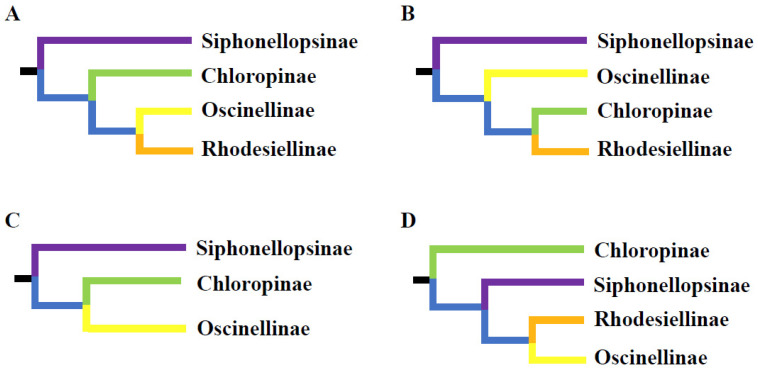
Phylogenetic relationships among subfamilies of the Chloropidae based on morphological data. (**A**,**B**). Nartshuk and Andersson, 2013 [15]; (**C**). Andersson, 1977 [25], Mlynarek and wheeler, 2018 [21]; (**D**). Bazya, 2019 [27].

The subfamily-level classification of Chloropidae has been widely discussed over recent decades (Figure 1). Members of Chloropidae are currently assigned to three or four subfamilies. Andersson [17] established a classification into three subfamilies—Siphonellopsinae, Chloropinae, and Oscinellinae—and described the tribe Rhodesiellini in the Oscinellinae. This division was accepted by Kanmiya [3], Sabrosky [18], Wheeler [19], Ismay and Nartshuk [20], Mlynarek and Wheeler [21], and Ismay et al. [4]. Nartshuk [22,23] treated Rhodesiellinae as a subfamily, which was followed by Cherian [24], Nartshuk [1], Nartshuk and Andersson [15], and Riccardi and Amorim [2], resulting in the recognition of Chloropidae comprising four subfamilies. Andersson [12,25] initially intended to reconstruct the phylogeny of the family. Due to high diversification and relatively recent evolution of chloropids, Andersson identified a considerable challenge in conducting a cladistic analysis of the family based on morphology. In his study, Andersson [25] analyzed general phylogenetic relationships within the family based on morphological data and concluded that Siphonellopsinae is the ‘basal subfamily’ (Figure 1). This is supported by most studies based on morphological data [2,3,23,26]. The relationships between Chloropinae, Oscinellinae, and Rhodesiellinae are, however, confusing. Nartshuk and Andersson [15] suggested either Chloropinae + (Oscinellinae + Rhodesiellinae) or Oscinellinae + (Chloropinae + Rhodesiellinae). Bazyar [27] suggested Chloropinae as the sister group of Siphonellopsinae + (Oscinellinae + Rhodesiellinae) (Figure 1). However, the Rhodesiellinae is poorly defined in his study, and the set of genera included in Rhodesiellinae corresponds to an early branching group of genera at the base of the Oscinellinae [27].

The dipteran mitochondrial genomes are relatively conservative in size and structure and rarely occur in gene rearrangement. Dipterans follow similar codon usage and nucleotide biases which are possibly influenced by mutational and selection pressures [28]. The mitochondrial genome phylogeny can largely reduce random phylogenetic errors arising from short genes or the high or low conservatism of individual genes [29]. Mitochondrial genomes have been widely used for phylogenetic inference for various taxonomic lineages of dipterans [30,31,32] because of their typically uniparental inheritance, lack of introns, high copy numbers, relatively simple structure, conserved gene composition, and rapid evolutionary rate [30]. However, limited attention has been given to individual chloropid mitochondrial genomes, with studies focusing primarily on *Anatrichus pygmaeus* [33], *Chlorops oryzae* [34], and *Dicraeus orientalis* [35]. This lack of data hinders a comprehensive phylogenetic analysis of Chloropidae. To elucidate the relationships among chloropid subfamilies, it is crucial and pressing to generate more mitochondrial genomes across the family and undertake comparative and phylogenetic analyses.

In this study, the mitochondrial genomes of 10 Chloropidae that cover all four subfamilies were newly generated and annotated, along with one Milichiidae outgroup. We integrated previously reported chloropid mitochondrial genomes; analyzed their genomic structures, nucleotide compositions, and substitutional and evolutionary rates; and reconstructed the phylogeny of the family. We aimed to (1) assess the validity of Rhodesiellinae and (2) to elucidate the subfamily-level relationships within Chloropidae.

## 2. Results

### 2.1. General Features and Genome Organization

**Figure 2:**
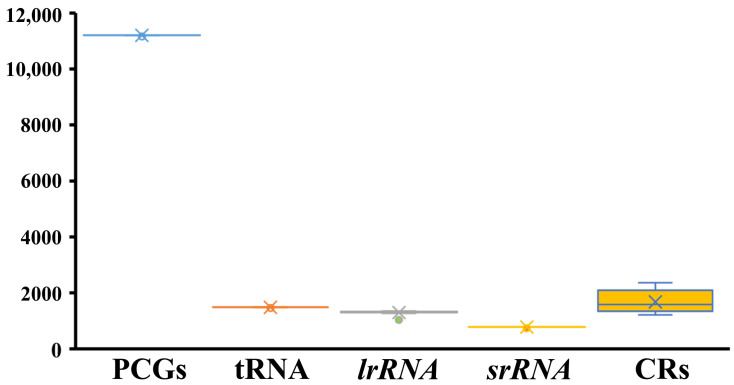
Sizes of the protein-encoding genes (PCGs), tRNAs, large ribosomal RNA (lrRNA), small ribosomal RNA (srRNA), and control region in chloropid mitochondrial genomes.

The mitochondrial genomes of ten chloropid species were successfully sequenced, resulting in five complete genomes: *Apotropina* sp.1, *Elachiptera insignis*, *Rhodesiella* sp., *Rhodesiella elegantula*, and *Thaumatomyia glabra*. These mitochondrial genomes are typically double-stranded circular molecules containing 37 genes (13 protein-coding genes, 2 rRNA genes, and 22 tRNA genes) and a control region (CR). Together with three previously available data, eight complete chloropid mitochondrial genomes were then utilized for our comparative analysis. The length of chloropid mitochondrial genomes ranged from 16,033 bp (*Apotropina* sp. 1) to 17,313 bp (*Chlorops oryzae*). The lengths of PCGs, tRNAs, and rRNAs for Chloropidae are nearly identical. However, significant size variation was observed in the control region (Figure 2). The majority strand (J-strand) encodes 23 genes, including 9 PCGs and 14 tRNAs, while the remaining 14 genes (4 PCGs, 8 tRNAs, and 2 rRNAs) are transcribed from the minority strand (N-strand). The gene order in chloropid mitochondrial genomes aligns with that of previously published mitochondrial genomes of Schizophora, demonstrating high conservation in Brachycera.

### 2.2. Base Composition

**Figure 3:**
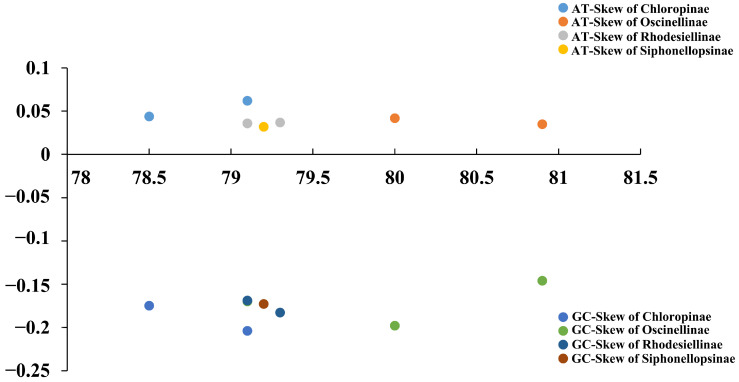
AT content (%), AT-Skew, and CG-Skew of 13 chloropid mitochondrial genomes.

The Chloropidae mitochondrial genomes exhibit a noticeable A+T bias, ranging from 78.5% (*Thaumatomyia glabra*) to 80.9% (*Elachiptera insignis*). In the PCGs, the base composition of each codon shows that the third codon positions have much higher A + T content compared to the first and second codon positions. All Chloropidae mitochondrial genomes show a positive AT-Skew and a negative GC-Skew (Figure 3).

### 2.3. Protein-Coding Genes, Codon Usage, and Evolutionary Rate Analysis

The total length of the 13 PCGs in the 14 Chloropidae flies ranges from 11,170 bp (*Dicraeus orientalis*) to 11,252 bp (*Pachylophus* sp.). *ATP8* is the shortest gene, while the largest gene is *ND5*. Most of the 13 PCGs start with the standard start codon ATN; however, in some cases, this pattern does not hold. For instance, *COI* of *Cetema* sp., *Chlorops oryzae*, and *Meromyza saltatrix* starts with ACG, and that of *Anatrichus pygmaeus*, *Apotropina* sp. 1, *Apotropina* sp. 2, *Cadrema minor*, *Dicraeus orientalis*, *Elachiptera insignis*, *Oscinella pusilla*, *Rhodesiella* sp., and *Rhodesiella elegantula* starts with TCG. Additionally, the *COII* of *Rhodesiella* sp. starts with GTG; the *ND1* of *Apotropina* sp. 2, *Cadrema minor*, *Elachiptera insignis*, *Meromyza saltatrix*, *Oscinella pusilla*, *Rhodesiella* sp., *Rhodesiella elegantula*, and *Thaumatomyia glabra* starts with TTG; and the start codon for *ND5* is GTT in *Cetema* sp. and CTC in *Meromyza saltatrix*. All PCGs end with TAA, TAG, or truncated termination codons such as TA or single T. The relative synonymous codon usage (RSCU) of mitogenomes across all subfamilies was calculated (Figure S1). *Ser2* and *Leu2* are the two most frequently utilized amino acids in Chloropidae.

To better comprehend the evolutionary rate of PCGs, we analyzed the mean ratios (ω) of non-synonymous to synonymous substitutions of the 13 PCGs to represent the selection pressures. Most of the ω values of PCGs are less than 1, except for the *ATP8* of some species (*Apotropina* sp.2: 1.10, *Cadrema minor*: 1.11, *Cetema* sp.1: 1.46, and *Pachylophus* sp.1: 1.20), which may be due to its sequence being too short (Figure S2). This result suggested that these protein-coding genes are evolving under a purifying selection. Within these PCGs, on average, *ATP8* (with ω = 0.89) demonstrates a relatively high value, suggesting it has undergone relaxed selection. By contrast, *COI* possesses the lowest Ka/Ks ratio (with ω = 0.07) and exhibits a strong purifying selection.

**Figure 4:**
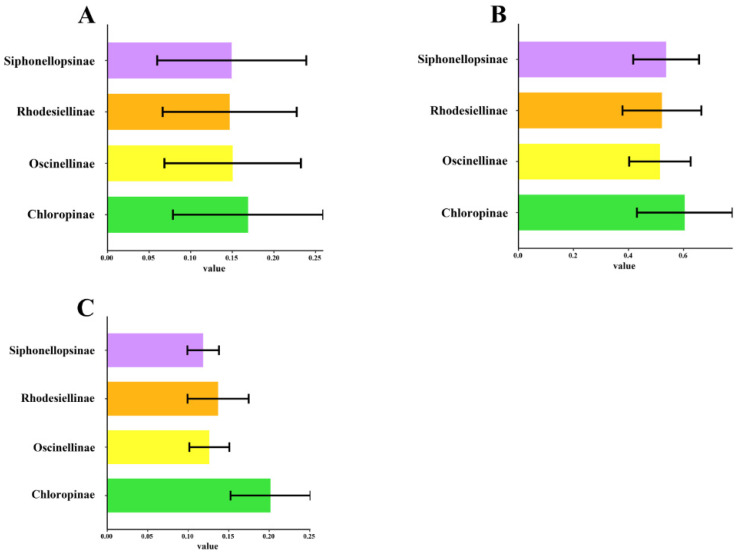
Subfamily-specific substitutions of 13 mitochondrial protein-coding genes in Chloropidae. (**A**) Average non-synonymous substitution rates (Ka) ± s.d. among four subfamilies; (**B**) average synonymous substitution rates (Ks) ± s.d. among four subfamilies; (**C**) pairwise distances ± s.d. of all 13 PCGs within subfamilies.

The subfamily Chloropinae exhibits a higher substitution value than Oscinellinae, Siphonellopsinae, and Rhodesiellinae (Figure 4). High substitution accumulation and faster changes in the genetic sequence suggest that the species has undergone significant genetic changes in a relatively short time, indicating potentially rapid diversification [36]. In contrast to the other three subfamilies, Chloropinae exhibits higher base substitution rates and genetic distances, implying that they may have undergone rapid evolution.

### 2.4. Intergenic Sequences

**Figure 5:**
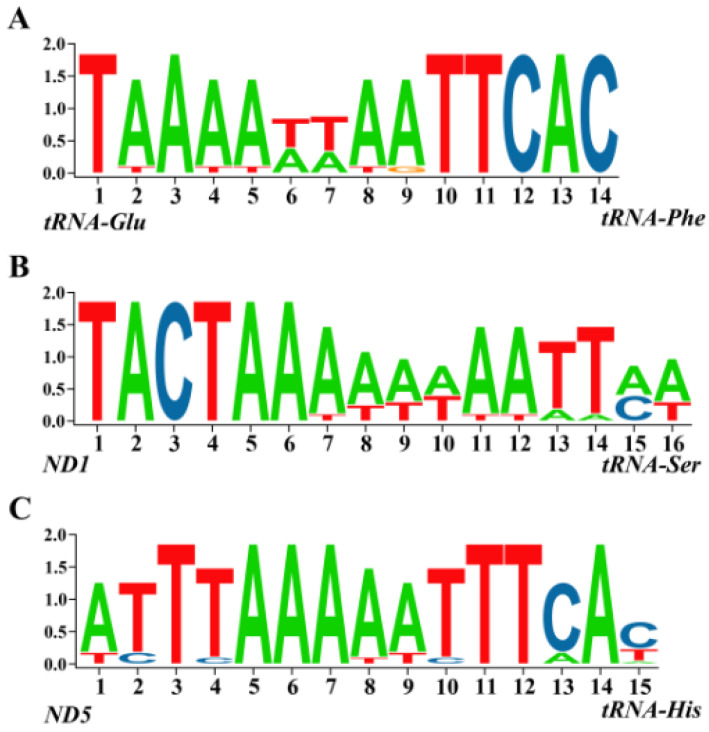
Nucleotide usage of three conserved intergenic sequences of 13 chloropid mitochondrial genomes. (**A**) Sequences between *tRNA^Glu^* and *tRNA^Phe^* (forward sequences); (**B**) sequences between *ND1* and *tRNA^Ser^* ^(*UCN*)^ (reversed sequences); (**C**) sequences between *ND5* and *tRNA^His^* (reversed sequences).

Previous studies on mitochondrial genomes of Cyclorrhapha have identified three conserved intergenic spacers: 18 bp between *ND1* and *tRNA^Ser (UCN)^*, 18 bp between *tRNA^Glu^* and *tRNA^Phe^*, and 15 bp between *tRNA^His^* and *ND5* [31,32]. All three of these conserved intergenic spacers were detected in chloropid mitochondrial genomes. Specifically, the spacer between *tRNA^Glu^* and *tRNA^Phe^* contains a 14 bp conserved sequence across all 14 examined chloropid flies; the spacer between *ND1* and *tRNA^Ser (UCN)^* exhibits a 16 bp conserved sequence, and the spacer between *ND5* and *tRNA^His^* shows a 15 bp conserved sequence. Conserved intergenic sequence blocks are presented in Figure 5.

### 2.5. Phylogenetic Analyses

**Figure 6:**
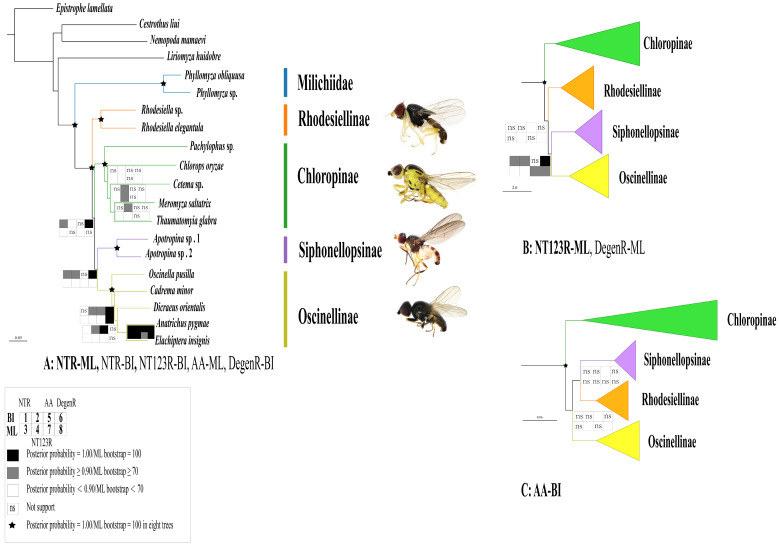
Phylogenetic trees of Chloropidae based on mitochondrial genome data. (**A**) Topology from ML inferences based on the NTR dataset showing relationships consistent with those recovered by NTR-BI, NT123R-BI, AA-ML, and DegenR-BI; (**B**) topology from ML inferences based on NT123R dataset showing relationships consistent with those recovered by DegenR-ML; (**C**) topology from BI based on the AA dataset. Squares at the nodes represent Bayesian posterior probabilities for 1, 2, 5, and 6 and ML bootstrap values for 3, 4, 7, and 8. The dataset of NTR corresponds to 1, 3 and 2, 4, AA to 5 and 7, DegenR to 6 and 8. A black square indicates posterior probabilities of 1.00 or an ML bootstrap of 100; a grey square indicates posterior probabilities between 0.90 and less than 1.00, or an ML bootstrap between 70 and less than 100; a white square indicates posterior probabilities less than 0.90, or an ML bootstrap less than 70; “ns” indicates a lack of support. Additionally, an asterisk is used to indicate posterior probabilities of 1.00 or ML bootstraps of 100 in all eight trees.

The phylogenetic tree obtained from Bayesian and maximum likelihood (ML) analyses yielded similar topological structures across all four datasets, and the majority of the nodes are robustly supported. The monophyly of the Chloropidae, Rhodesiellinae, Siphonellopsinae, and Oseinellinae was consistently fully supported by all analyses (posterior probability = 1.00 for all datasets; ML bootstrap = 100 for all datasets), similar to the sister group relationship between Chloropidae and Milichiidae (Figure 6). This is consistent with previous phylogenetic studies [5,16].

Relationships among four chloropid subfamilies were not robustly resolved, and three different topologies were generated based on eight phylogenetic estimations (Figure 6). Rhodesiellinae was weakly to fully supported as the sister group of remainders of the family by five analyses (NTR-ML, NTR-BI, NT123R-BI, AA-ML, and DegenR-BI) (Figure 6A). The remaining three analyses (NT123R-ML, DegenR-ML, and AA-BI) recovered Chloropinae as the earliest branching lineage of Chloropidae with fully supports (Figure 6B).

The monophyly of Siphonellopsinae + Oscinellinae was consistently supported in all analyses except AA-BI. This relationship was robustly supported by BI analyses with the degenerated nucleotide dataset and moderately supported by nucleotide datasets regardless of the partitioning schemes, whereas the ML method consistently provided weak supports (Figure 6A,B). The AA-BI analysis proposed Siphonellopsinae as the sister to Rhodesiellinae with weak supports (Figure 6C).

## 3. Discussion

In this study, we analyzed 14 chloropid mitochondrial genomes, including 10 newly sequenced genomes from four subfamilies. The nucleotide composition is highly biased towards A + T, ranging from 78.5% to 80.9%, which is similar to that of other dipteran flies [30,31]. Comparative analyses revealed that the chloropid mitogenomes are conserved in structure, which is consistent with all previously published mitochondrial genomes of Schizophora [31]. The 13 PCGs present multiple types of starting codons: the standard start codon (e.g., ATN) and no standard start codon (e.g., ACG, TCG, GTG, TTG, GTT, CTC). This result has also been found in previous studies [31]. The evolutionary rates of Chloropidae exhibit variation across subfamilies, with Chloropinae displaying a higher rate compared to the other three subfamilies, suggesting that Chloropinae may have undergone rapid evolution.

Three conserved intergenic sequences across Cyclorrhapha were found from all available chloropid mitochondrial genomes. Intergenic sequences serve multiple crucial roles in the genome. They contain regulatory elements such as promoters, enhancers, and transcription factor binding sites, which play a role in regulating gene expression [37]. The non-coding region situated between *ND1* and *tRNA^Ser (UCN)^* serves as the binding site for MtTERM, a highly conserved 7 bp motif across insects [38]. MtTERM regulates the expression levels of the rRNA genes relative to the protein-coding genes [37,39]. Furthermore, intergenic sequences are also important resources for the study of species evolution and phylogeny [40].

The subfamily-level relationships of Chloropidae have long been a topic of significant controversy across different phylogenetic analyses [3,15,25,26,27]. In the present study, maximum likelihood and Bayesian inference conducted on four datasets (NTR, NTR123, AA, and DegenR) indicated the monophyly of Chloropidae and all four subfamilies, which supports the previous hypothesis of monophyly [22,23,27]. Initially proposed as a tribe of Oscinellinae [17], Rhodesiellinae was elevated to subfamily status by Nartshuk [22,23]. However, the validity of Rhodesiellinae as a subfamily was doubted, as it was demonstrated to be paraphyletic in relation to the Oscinellinae based on morphological characters [27]. Three analyses indicated Chloropinae as the earliest branching lineage of Chloropidae with strong supports, consistent with Bazya’s findings based on morphological characters [27]. Although some morphological studies also showed that Siphonellopsinae is a sister to the remaining Chloropidae [2,3,15], this relationship was never recovered in our analyses.

Our study provides the first comprehensive molecular-based subfamily-level phylogenetic estimates on Chloropidae. However, the subfamily relationships within Chloropidae remain unresolved. This could be due to our limited taxon sampling within the diverse group of Chloropidae and mitochondrial genomes may lack adequate phylogenetic signals for resolving these relationships. Future studies should consider incorporating a more comprehensive taxon and gene sampling to effectively address this issue.

## 4. Methods and Materials

### 4.1. Taxon Sampling and DNA Extraction

We newly sequenced mitochondrial genomes of 10 chloropid species representing all four subfamilies, as well as one Milichiidae outgroup (Table 1). Adult flies were collected by net-sweeping in the field and preserved in 100% ethanol at −20 °C before DNA extraction. Specimens were identified based on morphological characteristics by Xiaoyan Liu using keys in Kanmiya [3], Yang and Yang [41], and Nartshuk and Andersson [15]. The genomic DNA was extracted from the thoracic muscle tissues of one specimen for each species using the DNeasy DNA Extraction kit (QIAGEN, Hilden, Germany). The remaining body parts of the sampled specimens were preserved as vouchers and deposited in the Hubei Insect Resources Utilization and Sustainable Pest Management Key Laboratory at Huazhong Agricultural University, Wuhan, China. Specimen collection information and associated voucher numbers are listed in Table 1 and Table S1.

### 4.2. Mitochondrial Genome Sequencing and Assembly

DNA samples were pooled for next-generation sequencing library construction following Gillett et al. [42]. The library was sequenced on the Illumina NovaSeq 6000 platform, generating 150 bp paired-end reads, by Novogene CO., LTD. (Beijing China). The raw reads were filtered and trimmed using Fastp [43]. De novo assemblies of high-quality reads were conducted using IDBA-UD [44] with a similarity threshold of 98% and minimum and maximum *k* values of 40 and 140 bp, respectively. The bait sequence COI was amplified by standard PCR reactions using universal primers (LepF: ATTCAACCAATCATAAAGATATTGG, LepR: TAAACTTCTGGATGTCCAAAAAATCA) as in Hajibabaei et al. [45], and a BLAST search was carried out with BioEdit 7.0.5.3 to identify the best-fit mitochondrial contigs [46].

### 4.3. Bioinformatic Analysis

Gene sequences were initially annotated by the MITOS Web Server (http://mitos2.bioinf.uni-leipzig.de/index.py (accessed on 3 January 2022)) [47]. Subsequently, PCGs and rRNA genes were manually modified by aligning with *Dicraeus orientalis* in Geneious. The locations of tRNA genes were confirmed by ARWEN 1.2 (http://mbio-serv2.mbioekol.lu.se/ARWEN/ (accessed on 3 January 2022)) [48]. The nucleotide composition of mitochondrial genomes and relative synonymous codon usage (RSCU) values of each PCG were analyzed using PhyloSuite v1.2.3 [49]. Genomic DNA compositional differences between genes were measured using AT-skew [(A − T)/(A + T)] and GC-skew [(G − C)/(G + C)]. KaKs_Calculator 2.0 was utilized to calculate the non-synonymous (Ka) and synonymous (Ks) substitution rates of the PCGs [50].

**Table 1:** Information of samples for the phylogenetic analyses used in the study.

Family	Subfamily	Species	Accession Number	Reference
**Outgroup**				
Syrphidae	Syrphinae	*Epistrophe lamellata*	MZ398236	[51]
Sepsidae	Sciomyzoidea	*Nemopoda mamaevi*	KM605250	[31]
Lauxaniidae		*Cestrotus liui*	KX372559	[32]
Agromyzidae	Phytomyzinae	*Liriomyza huidobrensis*	JN570505	[52]
Milichiidae		*Phyllomyza* sp.	OP612805	this study
		*Phyllomyza obliquusa*	MT462165	[16]
**Ingroup**				
	Siphonellopsinae	*Apotropina* sp.1	OP612811	this study
		*Apotropina* sp.2	OP612809	this study
Chloropidae	Chloropinae	*Cetema* sp.	OR522694	this study
		*Chlorops oryzae*	NC_059894	[34]
		*Meromyza saltatrix*	NC_072204	this study
		*Pachylophus* sp.	MT462163	[16]
		*Thaumatomyia glabra*	NC_072209	this study
	Rhodesiellinae	*Rhodesiella* sp.	OP612804	this study
		*Rhodesiella elegantula*	NC_072206	this study
	Oscinellinae	*Anatrichus pygmaeus*	NC_063616	[33]
		*Cadrema minor*	NC_072207	this study
		*Dicraeus orientalis*	NC_057210	[35]
		*Elachiptera insignis*	NC_072208	this study
		*Osciella pusilla*	NC_072205	this study

### 4.4. Phylogenetic Analysis

Twenty mitochondrial genomes were employed for phylogenetic analysis. Ten newly sequenced and four GenBank-available chloropid mitochondrial genomes were used as ingroups, covering all four recognized subfamilies (Table 1). Sequences from Syrphidae, Sepsidae, Lauxaniidae, Agromyzidae, and Milichiidae were selected as outgroup taxa (Table 1).

PhyloSuite v1.2.3 was used from mitochondrial genome extraction to matrix preparation [49]. Each PCG and rRNA was aligned using the MAFFT module under the ‘--auto’ strategy [53]. All ambiguously aligned sites were removed using trimAl [54]. Alignments of individual genes were concatenated to build different datasets, and TreeSuite [55] was used to evaluate their saturations (Figures S3–S6). Four datasets and partitioning scheme combinations were prepared for phylogenetic analyses: (1) NTR, consisting of 13 PCGs and 2 rRNAs, partitioned by genes (15 partitions) consisting of 13,200 residues; (2) NT123R, consisting of 13 PCGs and 2 rRNAs, partitioned by genes and codon positions (41 partitions) consisting of 13,200 residues; (3) AA, consisting of amino acid sequences of 13 PCGs, partitioned by genes (13 partitions) consisting of 13,200 residues; (4) DegenR, consisting of ‘degenerated’ 13 PCGs and 2 rRNAs, partitioned by genes (15 partitions) consisting of 13,200 residues. The degenerated PCGs were generated using the Degen script [56,57], wherein all synonymous sites were reassigned according to the IUPAC ambiguity nomenclature.

Phylogenetic trees were constructed under maximum likelihood (ML) methods and Bayesian inference (BI). ML analyses were carried out using IQ-TREE v.2.1.3 [58]. Datasets were partitioned and model-tested in ModelFinder [59] as implemented in IQ-TREE. We found the best partition scheme after merging possible partitions (‘-MFP+MERGE’ command) and determining the best scheme under the Bayesian information criterion (BIC). The best-fitting models were used for phylogenetic reconstructions (‘-p’ command). An initial 1000 parsimony trees were generated in IQ-TREE with the command ‘-ninit 1000′, and the 100 trees with the fewest steps were used to initialize the candidate set (-ntop 100), considering all possible nearest neighbor interchanges (-allnni). These 100 trees were maintained in the candidate set during the ML tree search (-nbest 100), and unsuccessful runs were terminated after 1000 iterations (-nstop 1000). Perturbation strength was set to 0.2 (-pers 0.2), as recommended for datasets with many short sequences. We applied nearest-neighbor interchange (NNI) branch swapping to improve the tree search and limit overestimating branch supports due to severe model violations (‘-bnni’ command). Node supports were computed with 1000 UFBoot (‘-B’ command) replicates [60,61] and SH-aLRT (‘-alrt’ command) [62]. BI analysis was performed using MrBayes 3.2.7 [63]. PartitionFinder v2.1.1 was used to assess the optimal partitioning strategy and substitution model using the greedy algorithm and BIC criterion [64]. Two independent runs were executed for 1–2 million generations, with sampling occurring every 1000 generations. Additionally, four independent Markov Chain Monte Carlo (MCMC) chains were employed, consisting of three heated chains and a cold chain, and the initial 25% of samples were discarded as burn-in. When the average standard deviation of split frequencies fell below 0.01, we considered that stationarity had been reached. The phylogenetic trees generated in this study were visualized using Figtreev1.4.4 (http://tree.bio.ed.ac.uk/software/figtree/ (accessed on 25 November 2018)).

Branches were considered fully supported if SH-aLRT = 100 AND UFBoot = 100 for ML, pp = 1.0 for BI; robustly supported if SH-aLRT ≥ 80 AND UFBoot ≥ 95 for ML, pp ≥ 0.9 for BI; moderately supported if SH-aLRT ≥ 80 OR UFBoot ≥ 95 for ML, 0.9 ≥ pp ≥ 0.8 for BI; and weakly supported if SH-aLRT < 80 AND UFBoot < 95 for ML, pp < 0.8 for BI.

## 5. Conclusions

This study presents the first comprehensive comparative analysis of mitochondrial genomes for Chloropidae, providing valuable insights into the phylogeny and evolution of this family. The comparative analysis revealed that Chloropidae have a gene arrangement that is identical to other dipterans. Furthermore, three conserved intergenic sequence blocks were identified (*ND1* and *tRNA^Ser (UCN)^*, *tRNA^Glu^* and *tRNA^Phe^*, *tRNA^His^* and *ND5*) in the mitochondrial genomes. Evolutionary rates within Chloropidae vary significantly across subfamilies, with Chloropinae exhibiting higher rates than the other three subfamilies. Moreover, the phylogenetic results supported the monophyly of Chloropidae but failed to construct a well-supported hypothesis regarding the phylogenetic relationships between four subfamilies, possibly due to rapid evolution in grass flies.

## Data Availability

The mitochondrial genomes newly generated in this study have been deposited in GenBank (OP612804, OP612805, OP612809, OP612811, OR522694, NC072204-NC072209).

## Supplementary Materials

The following supporting information can be downloaded at: https://www.mdpi.com/article/10.3390/ijms25052920/s1.

## References

[1] Nartshuk E.P. (2012). A check list of the world genera of the family Chloropidae (Diptera, Cyclorrhapha, Muscomorpha). Zootaxa.

[2] Riccardi P.R., Amorim D.D.S. (2020). Phylogenetic relationships and classification of the Chloropinae of the world (Diptera: Chloropidae). Zool. J. Linn. Soc..

[3] Kanmiya K. (1983). A systematic study of the Japanese Chloropidae (Diptera). Mem. Ent. Soc. Wash..

[4] Ismay J.W., Ismay B., Deeming J.C., Kirk-Spriggs A.H., Sinclair B.J. (2021). 96. Chloropidae. Manual of Afrotropical Diptera. Volume 3. Brachycera-Cyclorrhapha, Excluding Calyptratae.

[5] Wiegmann B.M., Trautwein M.D., Winkler I.S., Barr N.B., Kim J.W., Lambkin C., Bertone M.A., Cassel B.K., Bayless K.M., Heimberg A.M. (2011). Episodic radiations in the fly tree of life. Proc. Natl. Acad. Sci. USA.

[6] Evenhuis N.L. (1994). Catalog of the Fossil Flies of the World (Insecta: Diptera).

[7] Paganelli C.H.M., Sabrosky C.W. (1993). Hippelates flies (Diptera: Chloropidae) possibly associated with Brazilian purpuric fever. P. Entomol. Soc. Wash..

[8] Nartshuk E.P. (2014). Grass-fly larvae (Diptera, Chloropidae): Diversity, habitats, and feeding specializations. Entomol. Rev..

[9] Bower C., Towle B., Bickel D. (2015). Reproductive success and pollination of the Tuncurry midge orchid (*Genoplesium littorale*) (Orchidaceae) by chloropid flies. Telopea..

[10] Oelschlägel B., Nuss M., von Tschirnhaus M., Pätzold C., Neinhuis C., Dötterl S., Wanke S. (2015). The betrayed thief–the extraordinary strategy of *Aristolochia rotunda* to deceive its pollinators. New Phytol..

[11] Wiesenborn W.D. (2016). Conspecific pollen on insects visiting female flowers on the oak parasite *Phoradendron coryae* (Viscaceae). West. N. Am. Nat..

[12] Kidyoo A., Kidyoo M., McKey D., Proffit M., Deconninck G., Wattana P., Uamjan N., Ekkaphan P. (2022). Pollinator and floral odor specificity among four synchronopatric species of *Ceropegia* (Apocynaceae) suggests ethological isolation that prevents reproductive, Interference. Sci. Rep..

[13] Iwasa M., Oikawa S., Kanmiya K. (2013). *Siphunculina quinquangula* (Loew) (Diptera, Chloropidae) new to Japan: Emergence from the remains stage of pig carcass, with the implications for forensic entomology. Med. Entomol. Zool..

[14] Buck M. (2006). A new family and genus of acalypterate flies from the Neotropical region, with a phylogenetic analysis of Carnoidea family relationships (Diptera, Schizophora). Syst. Entomol..

[15] Nartshuk E.P., Andersson H. (2013). The frit flies (Chloropidae, Diptera) of Fennoscandia and Denmark. Fauna. Ent. Scand..

[16] Song N., Xi Y.Q., Yin X.M. (2022). Phylogenetic relationships of Brachycera (Insecta: Diptera) inferred from mitochondrial genome sequences. Zool. J. Linn. Soc..

[17] Andersson H. (1997). Taxonomic and phylogenetic studies on Chloropidae (Diptera) with special reference to Old World genera. Ent. Scand. Suppl..

[18] Sabrosky C.W. (1941). An Annotated List of Genotypes of the Chloropidae of the World (Diptera). Ann. Entomol. Soc. Am..

[19] Wheeler T.A., Brown B.V., Borkent A., Cumming J.M., Wood D.M., Woodley N.E., Zumbadoeds M.A. (2010). 92. Chloropidae (frit flies, grass flies, eye gnats). Manual of Central American Diptera.

[20] Ismay J., Nartshuk E.P., Papp L., Darvas B. (2000). Family Chloropidae. Contributions to a Manual of Palaearctic Diptera (with Special Reference to Flies of Economic Importance). Appendix volume.

[21] Mlynarek J.J., Wheeler T.A. (2018). Phylogeny and revised classification of the tribe Elachipterini (Diptera: Chloropidae). Zootaxa.

[22] Nartshuk E.P. (1984). Classification of the superfamily Chloropoidea (Diptera, Cyclorrhapha). Entomol. Rev..

[23] Nartshuk E.P. (1987). Chloropid flies (Diptera: Chloropoidea): Their system, evolution and association with plants. Trudy. Zool. Inst..

[24] Cherian P.T. (2002). Chloropidae (Part 1). Siphonellopsinae and Rhodesiellinae. The Fauna of India and Adjacent Countries. Diptera Volume IX.

[25] Andersson H. (1979). Problem vid kladistik analys av flugfamiljen Chloropidae. Ent. Tidskr..

[26] Brake I. (2000). Phylogenetic systematics of the Milichiidae (Diptera, Schizophora). Entomol. Scand..

[27] Bazyar Z.A. (2019). Comparative Morphology of Oscinellinae Genera (Diptera: Chloropidae). Ph.D. Thesis.

[28] Ramakodi M.P., Singh B., Wells J.D., Guerrero F., Ray D.A. (2015). A 454 sequencing approach to dipteran mitochondrial genome research. Genomics.

[29] Holland B.R., Jermiin L.S., Moulton V. (2005). Improved consensus network techniques for genome-scale phylogeny. Mol. Biol. Evol..

[30] Zhang X., Kang Z.H., Ding S.M., Wang Y.Y., Borkent C., Saigusa T., Yang D. (2019). Mitochondrial Genomes Provide Insights into the Phylogeny of Culicomorpha (Insecta: Diptera). Int. J. Mol. Sci..

[31] Li X.K., Ding S.M., Cameron S.L., Kang Z.H., Wang Y.Y., Yang D. (2012). The first mitochondrial genome of the sepsid fly *Nemopoda mamaevi* Ozerov, 1997 (Diptera: Sciomyzoidea: Sepsidae), with mitochondrial genome phylogeny of cyclorrhapha. PLoS ONE.

[32] Li X.K., Li W.L., Ding S.M., Cameron S.L., Mao M., Shi L., Yang D. (2017). Mitochondrial Genomes Provide Insights into the Phylogeny of Lauxanioidea (Diptera: Cyclorrhapha). Int. J. Mol. Sci..

[33] Cai X.D., Yang D., Liu X.Y. (2022). The complete mitochondrial genome of *Anatrichus pygmaeus* Lamb, 1918 (Diptera, Chloropidae). Mitochondrial DNA B.

[34] Wang J., Li X.Y., Du R.B., Liu Y.H. (2021). The complete mitogenome of *Chlorops oryzae* Matsumura (Diptera: Chloropidae). Mitochondrial DNA B.

[35] Liu J.Z., Li X., Cai X.D., Du B.T., Liu X.Y., Yang D. (2021). The complete mitochondrial genome of *Dicraeus orientalis* Becker, 1911 (Diptera: Chloropidae). Mitochondrial DNA B.

[36] Yan L.P., Xu W.T., Zhang D., Li J.Q. (2021). Comparative analysis of the mitochondrial genomes of flesh flies and their evolutionary implication. Int. J. Biol. Macromol..

[37] Roberti M., Polosa P.L., Bruni F., Musicco C., Gadaleta M.N., Cantatore P. (2003). DmTTF, a novel mitochondrial transcription termination factor that recognizes two sequences of Drosophila melanogaster mitochondrial DNA. Nucleic Acids Res..

[38] Cameron S.L., Whiting M.F. (2007). Mitochondrial genomic comparisons of the subterranean termites from the Genus *Reticulitermes* (Insecta: Isoptera: Rhinotermitidae). Genome.

[39] Taanman J.W. (1999). The mitochondrial genome: Structure, transcription, translation and replication. Biochim. Biophys. Acta Bioenerg..

[40] Lee C., Wen J. (2004). Phylogeny of Panax using chloroplast trnC-trnD intergenic region and the utility of trnC-trnD in interspecific studies of plants. Mol. Phylogenet. Evol..

[41] Yang D., Yang C.K., Xue W.Q., Chao C.M. (1998). Chloropidae of China (Diptera). Flies of China 1.

[42] Gillett C.P., Crampton-Platt A., Timmermans M.J., Jordal B.H., Emerson B.C., Vogler A.P. (2014). Bulk de novo mitogenome assembly from pooled total DNA elucidates the phylogeny of weevils (Coleoptera: Curculionoidea). Mol. Biol. Evol..

[43] Chen S.F., Zhou Y.Q., Chen Y., Gu J. (2018). fastp: An ultra-fast all-in-one FASTQ preprocessor. Bioinformatics.

[44] Peng Y., Leung H.C.M., Yiu S.M., Chin F.Y.L. (2012). IDBA-UD: A de novo assembler for single-cell and metagenomic sequencing data with highly uneven depth. Bioinformatics.

[45] Hajibabaei M., Janzen D.H., Burns J.M., Hallwachs W., Hebert P.D. (2006). DNA barcodes distinguish species of tropical Lepidoptera. Proc. Natl. Acad. Sci. USA.

[46] Alzohairy A.M. (2011). Bioedit: An important software for molecular biology. GERF Bull. Biosci..

[47] Donath A., Jühling F., Al-Arab M., Bernhart S.H., Reinhardt F., Stadler P.F., Middendorf M., Bernt M. (2019). Improved annotation of protein-coding genes boundaries in metazoan mitochondrial genomes. Nucleic Acids Res..

[48] Laslett D., Canback B. (2008). ARWEN: A program to detect tRNA genes in metazoan mitochondrial nucleotide sequences. Bioinformatics.

[49] Zhang D., Gao F.L., Jakovlić I., Zou H., Zhang J., Li W.X., Wang G.T. (2020). PhyloSuite: An integrated and scalable desktop platform for streamlined molecular sequence data management and evolutionary phylogenetics studies. Mol. Ecol. Resour..

[50] Wang D., Zhang Y., Zhang Z., Zhu J., Yu J. (2010). KaKs_Calculator 2.0: A toolkit incorporating gamma-series methods and sliding window strategies. Genom. Proteom. Bioinf..

[51] Li H., Yan Y., Li J. (2023). Eighteen mitochondrial genomes of Syrphidae (Insecta: Diptera: Brachycera) with a phylogenetic analysis of Muscomorpha. PLoS One.

[52] Wang S.Y., Lei Z.R., Wen J.Z., Wang H.H., Li X., Dong B.X., Ren B.Z. (2014). The complete mitochondrial genome of *Liriomyza huidobrensis* and comparison with *L. trifolii* and *L.sativae* (Diptera: Agromyzidae). Mitochondrial DNA.

[53] Katoh K., Standley D.M. (2013). MAFFT multiple sequence alignment software version 7: Improvements in performance and usability. Mol. Biol. Evol..

[54] Capella-Gutiérrez S., Silla-Martínez J.M., Gabaldón T. (2009). trimAl: A tool for automated alignment trimming in large-scale phylogenetic analyses. Bioinformatics.

[55] Xiang C.Y., Gao F.L., Jakovlić I., Lei H.P., Hu Y., Zhang H., Zou H. (2023). Using PhyloSuite for molecular phylogeny and tree-based analyses. iMeta.

[56] Regier J.C., Shultz J.W., Zwick A., Hussey A., Ball B., Wetzer R., Martin J.W., Cunningham W.C. (2010). Arthropod relationships revealed by phylogenomic analysis of nuclear protein-coding sequences. Nature.

[57] Zwick A., Regier J.C., Zwickl D.J. (2012). Resolving discrepancy between nucleotides and amino acids in deep-level arthropod phylogenomics: Differentiating serine codons in 21-amino-acid models. PLoS ONE.

[58] Nguyen L.T., Schmidt H.A., von Haeseler A., Minh B.Q. (2015). IQ-TREE: A fast and effective stochastic algorithm for estimating maximum-likelihood phylogenies. Mol. Biol. Evol..

[59] Kalyaanamoorthy S., Minh B.Q., Wong T.K.F., von Haeseler A., Jermiin L.S. (2017). ModelFinder: Fast model selection for accurate phylogenetic estimates. Nat. Methods.

[60] Minh B.Q., Nguyen M.A., von Haeseler A. (2013). Ultrafast approximation for phylogenetic bootstrap. Mol. Biol. Evol..

[61] Hoang D.T., Chernomor O., von Haeseler A., Minh B.Q., Vinh L.S. (2018). UFBoot2: Improving the Ultrafast Bootstrap Approximation. Mol. Biol. Evol..

[62] Guindon S., Dufayard J.F., Lefort V., Anisimova M., Hordijk W., Gascuel O. (2010). New algorithms and methods to estimate maximum-likelihood phylogenies: Assessing the performance of PhyML 3.0. Syst. Biol..

[63] Ronquist F., Teslenko M., van der Mark P., Ayres D.L., Darling A., Hohna S., Larget B., Liu L., Suchard M.A., Huelsenbeck J.P. (2012). MrBayes 3.2: Efficient Bayesian phylogenetic inference and model choice across a large model space. Syst. Biol..

[64] Lanfear R., Frandsen P.B., Wright A.M., Senfeld T., Calcott B. (2017). PartitionFinder 2: New Methods for Selecting Partitioned Models of Evolution for Molecular and Morphological Phylogenetic Analyses. Mol. Biol. Evol..

