# Supplementary material for: Comparative Analysis of the Mitochondrial Genomes of Chloropidae and Their Implications for the Phylogeny of the Family
Source: Int J Mol Sci. 2024 Mar 2;25(5):2920. doi: 10.3390/ijms25052920 (PMC10932363; doi:10.3390/ijms25052920)
Supplement: Supplementary file 1 [file ijms-25-02920-s001.zip › ijms-2840974-supplementary.pdf]

Supplement Data

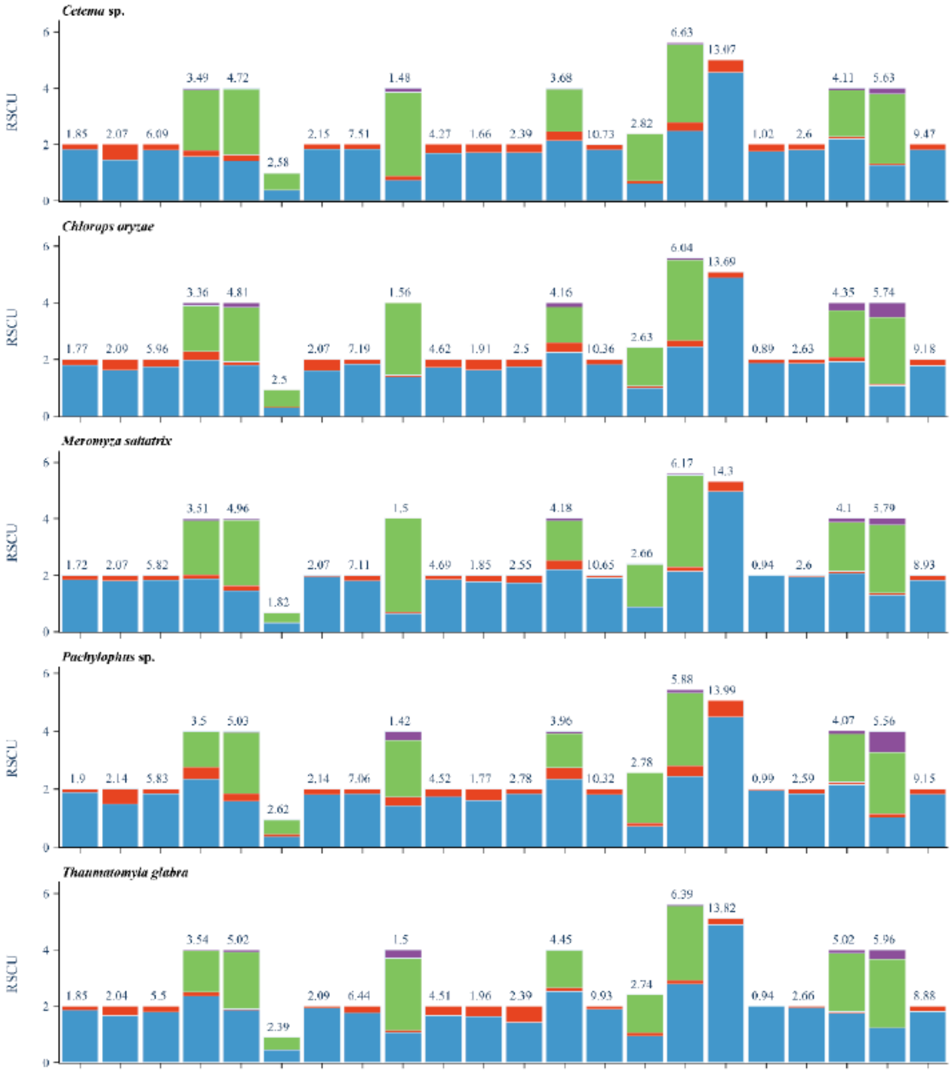

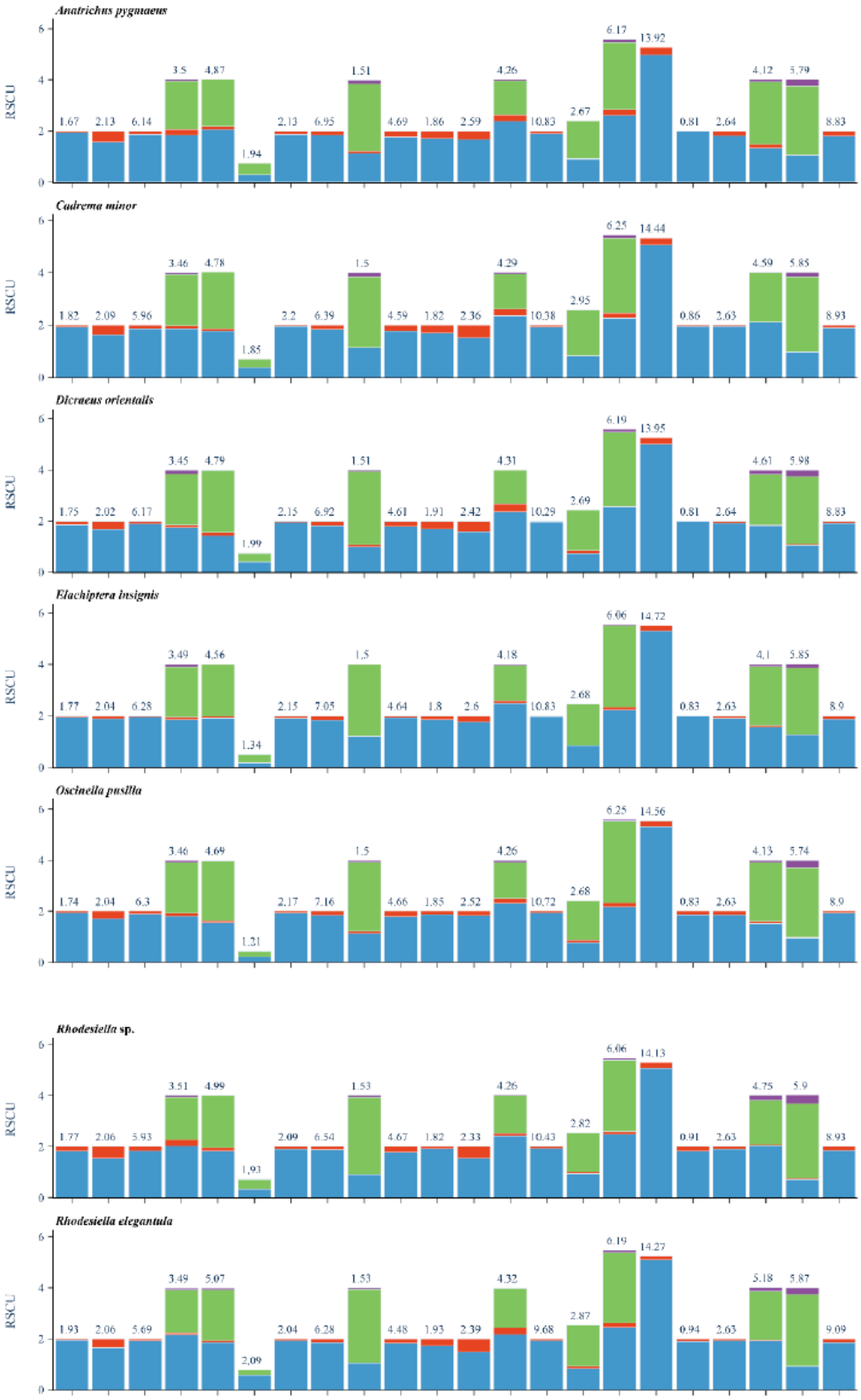
Anatrichus pygmaeus
RSCU
1.67 2.13 6.14 3.5 4.87 1.94 2.13 6.95 1.51 4.69 1.86 2.59 4.26 10.83 2.67 6.17 13.92 0.81 2.64 4.12 5.79 8.83
Cadrema minor
RSCU
1.82 2.09 5.96 3.46 4.78 1.85 2.2 6.39 1.5 4.59 1.82 2.36 4.29 10.38 2.95 6.25 14.44 0.86 2.63 4.59 5.85 8.93
Dicraeus orientalis
RSCU
1.75 2.02 6.17 3.45 4.79 1.99 2.15 6.92 1.51 4.61 1.91 2.42 4.31 10.29 2.69 6.19 13.95 0.81 2.64 4.61 5.98 8.83
Elachiptera insignis
RSCU
1.77 2.04 6.28 3.49 4.56 1.34 2.15 7.05 1.5 4.64 1.8 2.6 4.18 10.83 2.68 6.06 14.72 0.83 2.63 4.1 5.85 8.9
Oscinella pusilla
RSCU
1.74 2.04 6.3 3.46 4.69 1.21 2.17 7.16 1.5 4.66 1.85 2.52 4.26 10.72 2.68 6.25 14.56 0.83 2.63 4.13 5.74 8.9
Rhodesiella sp.
RSCU
1.77 2.06 5.93 3.51 4.99 1.93 2.09 6.54 1.53 4.67 1.82 2.33 4.26 10.43 2.82 6.06 14.13 0.91 2.63 4.75 5.9 8.93
Rhodesiella elegantula
RSCU
1.93 2.06 5.69 3.49 5.07 2.09 2.04 6.28 1.53 4.48 1.93 2.39 4.32 9.68 2.87 6.19 14.27 0.94 2.63 5.18 5.87 9.09

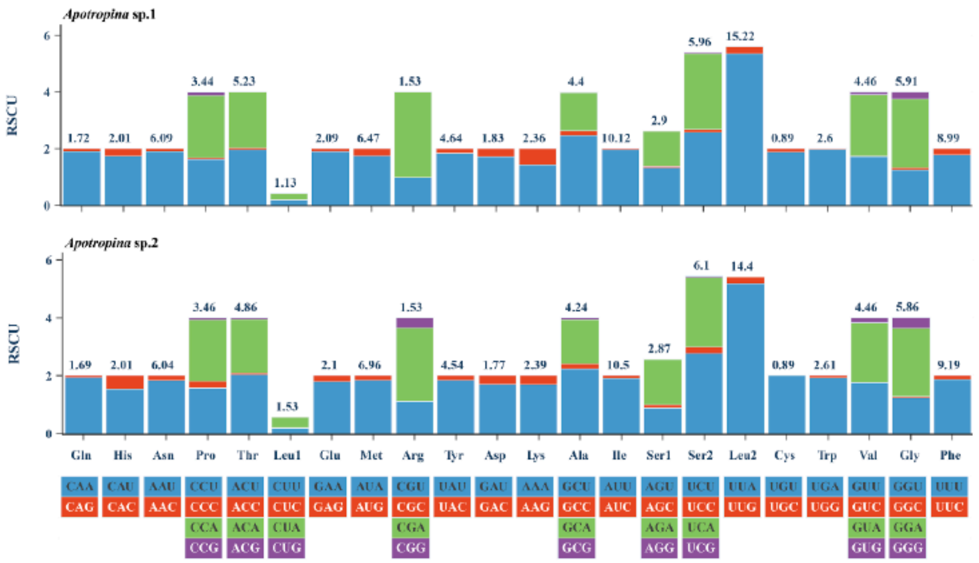

Figure S1. Relative synonymous codon usage in Chloropidae.

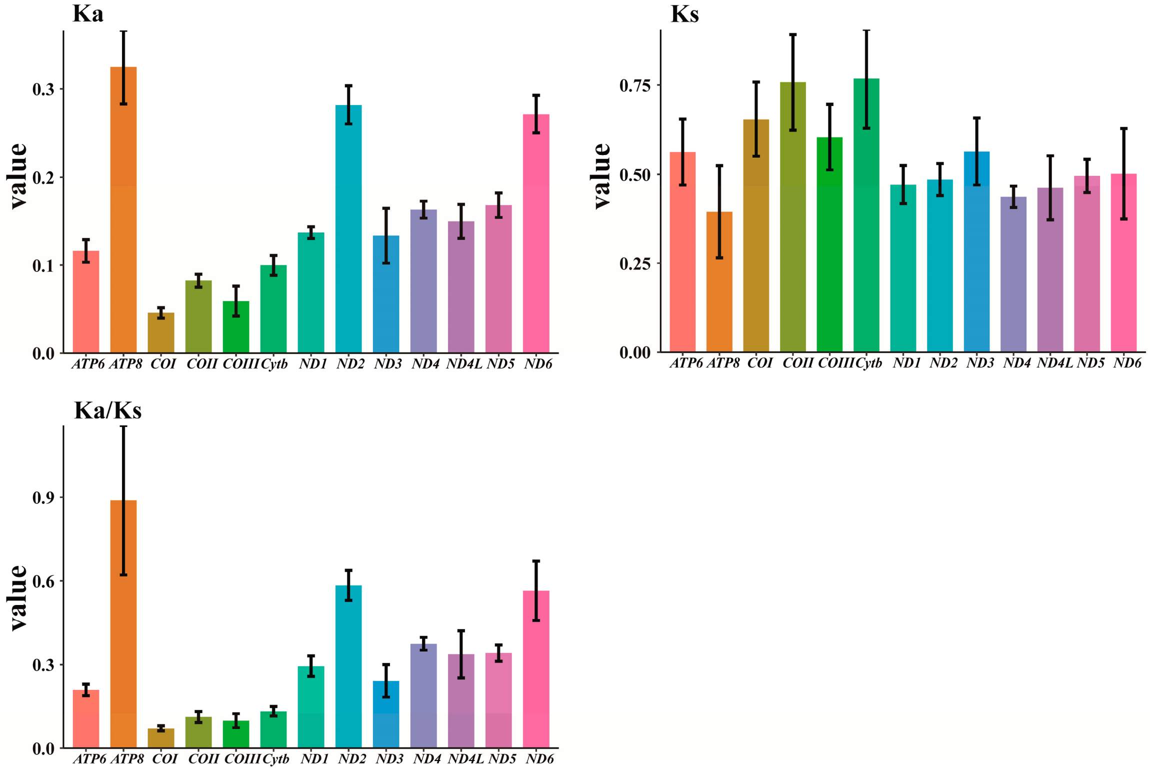

Figure S2. Nonsynonymous substitution rate (Ka), synonymous substitution rate (Ks) and Ka/Ks value ± s.d. of each subfamily.

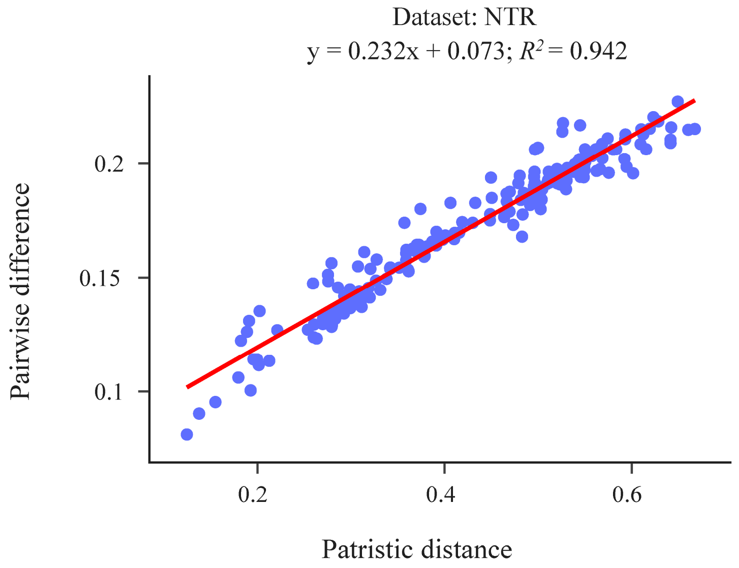

Figure S3. Saturation analysis of NTR dataset.

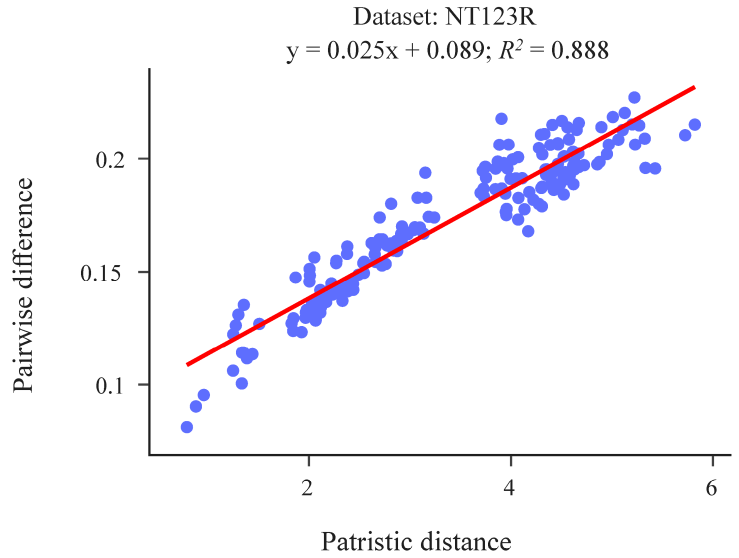

Figure S4. Saturation analysis of NT123R dataset.

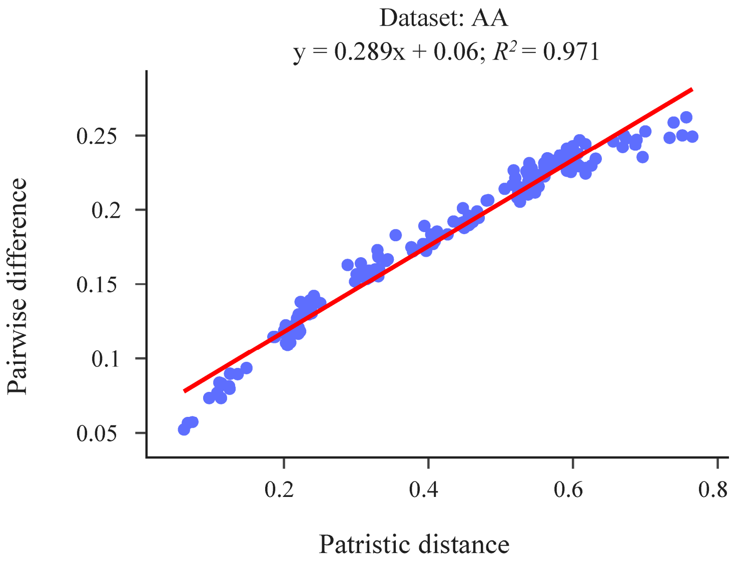

Figure S5. Saturation analysis of AA dataset.

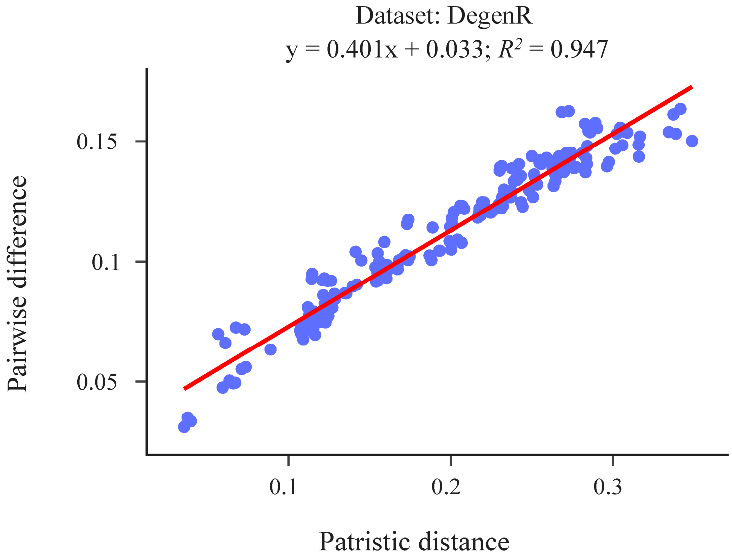

Figure S6. Saturation analysis of DegenR dataset.

Table S1. Detailed information on the specimens used in this study.

Figure .	Subfamily	species	Collect information
Chloropidae	Chloropinae	*Meromyza saltatrix*	Bashayar, Shufu, Xinjiang, China (39.36N, 75.46E), 2014.VII.7, Xuankun Li
	Oscinellinae	*Thaumatomyia glabra*	Talati, Qinghe, Xinjiang, China (43.25N, 93.40E), 2014.VI.26, Xuankun Li
		Cetema sp.	Baihaba, Habahe, Xinjiang, China (48.31N, 86.40E), 2014.VII.2, Xuankun Li
		Elachiptera insignis	Manwa, Xishuangbanna, Yunnan, China (22.05N 100.25E), 2020.Ⅺ.24, Liang Wang
		Osciella pusilla	Kuonikemai, Fuhai, Xinjiang, China (47.09N, 87.44E), 2014.VI.29, Xuankun Li
		Cadrema minor	Shanggang, Mengla, Yunnan, China (21.28N, 101.70E), 2019.VII.9, Qicheng Yang
	Rhodesiellinae	*Rhodesiella* sp.	Bashayaguo, Shufu, Xinjiang, China (39.36N, 75.46E), 2014.VII.9, Xuankun Li
		Rhodesiella elegantula	Nanbian, Lingshan, Guangxi, China (24.95N, 110.27E), 2021.VIII.7, Xiaodong Cai
	Siphonellopsinae	*Apotropina* sp. 1	Sharen, Mengla, Yunnan, China (21.72N, 101.54E), 2019.VII.7, Xiaoyan Liu
		Apotropina sp. 2	Sharen, Mengla, Yunnan, China (21.72N, 101.54E), 2019.VII.7, Qicheng Yang
Milichiidae		*Phyllomyza* sp.	Zhanggeicun, Yajiang, Sichuan, China (29.41N, 98.66E), 2020.VI.25, Liang Wang
